# Investigating the Antimicrobial Properties of Essential Oil Constituents and Their Mode of Action

**DOI:** 10.3390/molecules29174119

**Published:** 2024-08-30

**Authors:** Ilham Noui Mehidi, Abdenour Ait Ouazzou, Wafa Tachoua, Karim Hosni

**Affiliations:** 1 Natural Resources Valorization and Bioengineering Laboratory, University Benyoucef Benkhedda Algiers 1, Alger Centre 16000, Algeria; 2 Department of Nature and Life Sciences, Faculty of Sciences, Algiers 1 University—Benyoucef Benkhedda, 2 Rue Didouche Mourad, Alger Centre 16000, Algeria; 3 Laboratoire des Substances Naturelles, Institut National de Recherche et d’Analyse Physico-Chimique, Sidi Thabet 2020, Tunisia

**Keywords:** essential oils, sublethal injury, antibacterial activity, repair, molecular docking

## Abstract

Essential oils (EOs) and plant extracts, rich in beneficial chemical compounds, have diverse applications in medicine, food, cosmetics, and agriculture. This study investigates the antibacterial activity of nine essential oil constituents (EOCs) against *Escherichia coli*, focusing on the effects of treatment pH and biosynthetic requirements. The impact of EOCs on bacterial inactivation in *E. coli* strains was examined using both nonselective and selective culture media. Computer-assisted drug design (CADD) methods were employed to identify critical binding sites and predict the main binding modes of ligands to proteins. The EOCs, including citral, α-terpinyl acetate, α-terpineol, and linalool, demonstrated significant bacterial inactivation, particularly under acidic conditions. This study revealed that EOCs have an effect on the presence of sublethal damage to both the cytoplasmic membrane and the outer membrane in Gram-negative bacteria. Adding penicillin G to the repair medium prevents the recovery of sublethal injuries in *E. coli* treated with α-terpinyl acetate, α-terpineol, linalool, and citral, indicating that peptidoglycan synthesis is essential for recovering from these injuries. However, penicillin G did not hinder the recovery process of most sublethally injured cells treated with the other assessed EOCs. Molecular docking studies revealed the favorable binding interactions of α-terpinyl acetate, α-terpineol, linalool, and citral with the β-lactamase enzyme Toho-1, indicating their potential as effective antibacterial agents. The findings suggest that EOCs could serve as viable alternatives to synthetic preservatives, offering new strategies for combating antibiotic-resistant bacteria.

## 1. Introduction

Essential oils (EOs) and plant extracts are rich in beneficial chemical elements and have a wide range of applications in medicine, food, cosmetics, and agriculture [1,2,3,4]. These plant-derived treatments have played a critical role in preventing and treating a variety of diseases throughout history, underscoring their importance in the synthesis of natural medication [5]. These EOs are derived from a variety of sources, including spices, herbs, fruits, and flowers, and contain a wide range of constituents, with hydrocarbon monoterpenes being particularly prominent [6]. Essential oils (EOs) have antimicrobial properties that are dependent on their chemical composition and the number of single components. Many EOs are constitutively expressed by plants or can be synthesized as self-defense mechanisms in response to pathogens [7]. Vegetables, spices, and fruits with high levels of EOs are excellent sources of natural elements with activity against microorganisms of agricultural and health interest. Components such as carvacrol, thymol, limonene, and citral have been extensively studied for their antibacterial effects in food preservation [7,8,9,10,11].

As the desire for natural and safe food items has grown, essential oil constituents (EOCs), which have gained the Generally Recognized as Safe (GRAS) classification, have sparked widespread interest in their potential for food preservation. Their GRAS classification distinguishes them as promising alternatives to synthetic preservatives capable of increasing the shelf life of perishable foods by suppressing the growth of foodborne microorganisms [12,13]. However, completely realizing their potential requires a thorough understanding of their methods of action, which include a variety of ingredients such as citral [14], thymol, and carvacrol [10,11]. On the one hand, chemical preservatives have traditionally been used to inhibit microbial growth, but current research suggests that they may also work as inactivating agents [15]. However, there is limited research about the efficacy of hydrophobic EOCs in removing microbes under varied concentrations, ambient circumstances, and incubation temperatures or durations [16].

Research into the antimicrobial activity of EOs and their constituents has focused mainly on their antibacterial properties, often neglecting the specific mechanisms of action. Nevertheless, EOs or their constituents can attack multiple sites inside a bacterial cell to inactivate the bacteria. Molecular docking techniques have revealed a robust binding affinity between the oils’ components and enzymes, including isoleucyl-tRNA synthetase, DNA gyrase, and the penicillin-binding protein. For instance, thymol and viridoflorol showed significant binding affinity, suggesting potent antibacterial potential against multidrug-resistant strains [17]. A thorough study of the antibacterial capabilities of EOs and their constituents could reveal new applications in the food industry, aiding in minimizing the risks associated with antibiotic resistance, a growing problem due to the excessive use of antibiotics in medicine and livestock farming [18]. The recent surge in infections caused by multidrug-resistant bacteria (MDR) in recent years has heightened interest in the antibacterial activities of medicinal plants and their metabolites. ß-lactams, the most commonly used antibacterial drugs, target specific bacterial components such as DD-transpeptidases and the penicillin-binding proteins (PBPs). However, bacteria have developed resistance mechanisms against these drugs [18,19,20]. Notably, there are presently no published studies on the β-lactamase inhibitory abilities of hydrocarbon monoterpenes; therefore, the development of novel β-lactamase inhibitors could be a promising approach to enhancing the effectiveness of low-dose treatments and reducing bacterial resistance.

This study aimed to achieve the following: (i) investigate the inactivation of *E. coli* by EOCs, taking into account the pH of the treatment medium; and (ii) assess the biosynthetic requirements for repairing sublethal injuries caused by EOCs in bacterial envelopes to identify the affected structures. To that end, we investigated in silico ligand–protein docking using computer-assisted drug design (CADD) approaches, which can be used to identify key sites and predict the dominant binding mode(s) of a ligand and a protein with a known three-dimensional structure, and further proposed structural hypotheses about how the ligands inhibit the target.

## 2. Results

### 2.1. Microbial Inactivation by Essential Oil Constituents Relative to Treatment Medium pH

**Figure 1:**
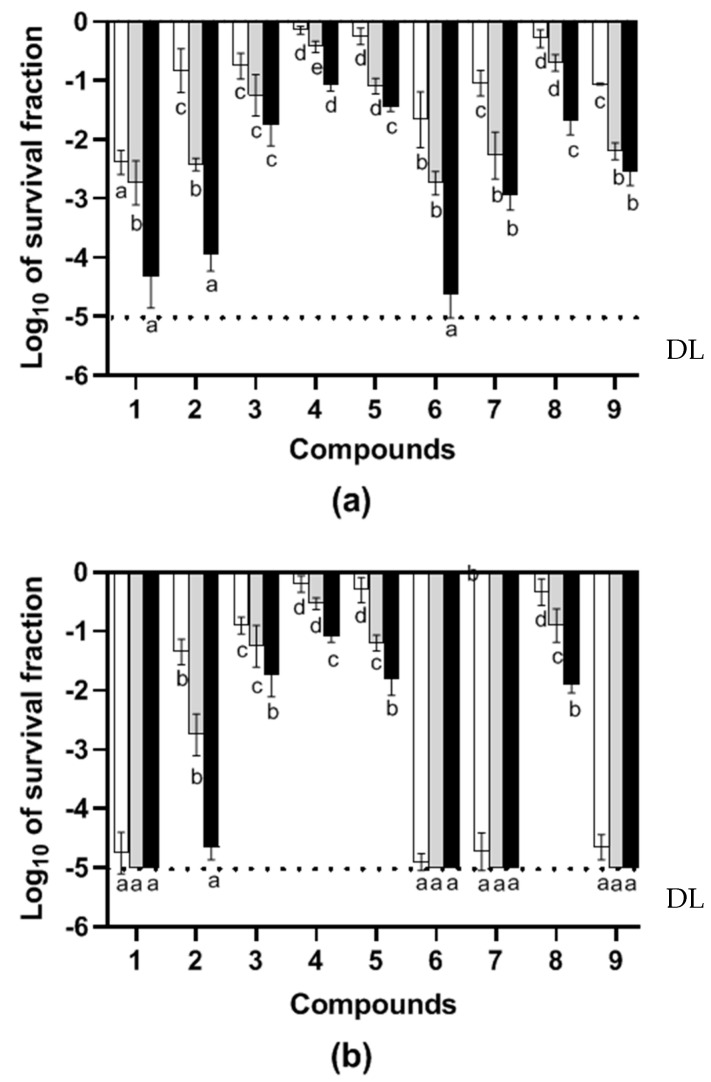
(**a**): Logarithmic survival fraction of *Escherichia coli* O157:H7 after 5 h treatment at room temperature in pH 7.0 citrate-phosphate buffer or (**b**): pH 4.0, in the presence of 0.2 μL/mL of the following pure constituents: α-terpinyl acetate (1), terpineol 4-ol (2), carvacrol (3), thymol (4), (+)-limonene (5), citral (6), linalool (7), 1,8-cineole (8), *p*-cymene (9). Survivors were recovered in nonselective TSAYE (☐), in selective medium added with sodium chloride (TSAYE-SC) (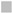), or selective medium added with bile salts (TSAYE-BS) (■). Data are mean ± standard deviation (error bars). The values with different superscripts (a, b, c, d, or e) in the same columns are significantly different (*p*< 0.05). DL: The dotted line represents the detection limit.

*E. coli* was chosen as the study’s representative Gram-negative bacteria. Monoterpenes were administered to the stationary phase cells at a dosage of 3 × 10^7^ CFU/mL. Treatment with 0.2 μL/mL of EOCs at pH 7.0 and 4.0 for 5 h resulted in significant inactivation, as shown in Figure 1. During a 5 h incubation, citral, α-terpineol, α-terpinyl acetate, linalool, and carvacrol significantly inhibited bacterial growth at pH 7.0. Notably, *E. coli* was more sensitive to these ingredients, resulting in sublethal damage to both the cytoplasmic and outer membranes. After 5 h, the entire analyzed population had sublethal damage, with about four log10 cycles of bacterial population decline. Other hydrocarbons, including limonene, thymol, *p*-cymene, and 1,8-cineole, induced less severe sublethal damage at pH 7.0 (Figure 1a).

At pH 4.0 (Figure 1b), several components were able to achieve five log10 cycles of *E. coli* inactivation after 5 h at ambient temperature, with *p*-cymene, carvacrol, thymol, and citral having the highest bactericidal activity. However, *E. coli* showed resistance to components such as limonene, thymol, *p*-cymene, and 1,8-cineole, attaining at least one log10 cycle of inactivation. Once again, the examination of survivors using the selected recovery media revealed that most components produced sublethal lesions, with outer membrane damage seeming to occur before cytoplasmic membrane injury. Most treated *E. coli* cells (more than 90%) absorbed propidium iodide in the presence of citral, carvacrol, thymol, α-terpinyl acetate, *p*-cymene, linalool, α-terpineol, (+)-limonene, and 1,8-cineole after 5 h at room temperature, indicating membrane permeabilization. A fluorescent microscope was used to visually detect the dye within the cells.

### 2.2. Biosynthetic Requirements for Restoring the Sublethal Injury Induced by EOCs

**Figure 2:**
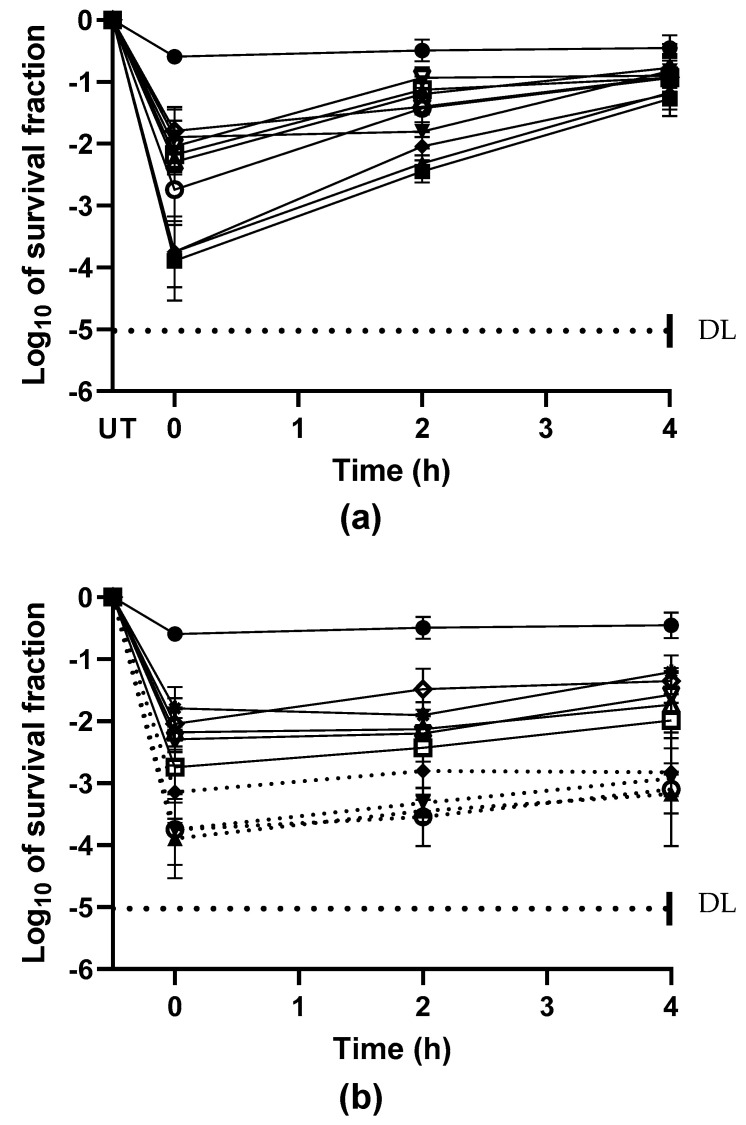
Biosynthetic requirements for the repair of sublethal injury caused in *E. coli* by exposure to essential oils constituents: α-terpinyl acetate (**∆**), terpineol 4-ol (**◊**), carvacrol (**▲**), thymol (**■**), (+)-limonene (**▼**), citral (**□**), Linalool (▽), 1,8-cineol (o), *p*-cymene (**♦**). Cells were exposed to 0.2 µL/L of EOCs for 3 h at pH 7.0, and then inoculated to subsequent incubation for 4 h at 30 °C in TSBYE (●,○) and into this medium containing penicillin G, (**a**) or without penicillin G (**b**). Survivors were recovered in nonselective medium (TSAYE) (●) and selective medium added with sodium chloride (TSAYE-SC). UT, untreated. Data represent mean ± standard deviation (error bars). DL: The dotted line represents the detection limit.

Figure 2 shows the surviving fraction of *E. coli* cells after treatment with EOCs, including citral, α-terpinyl acetate, α-terpinyl 4-ol, linalool, (+)-limonene, *p*-cymene, 1.8-cineole, thymol, and carvacrol with 0.2 μL/L (90 min; pH 7.0) (Figure 2a), and at pH4.0 (Figure 2b). Assessments were performed immediately following the treatment (time = 0) and after a 4 h incubation at 20 °C. At time = 0, half of the initial population perished, while more than 99% of the survivors had sublethal injuries. After a 240 min incubation in TSBYE, the injured population healed their membrane injuries and established colonies on the selective medium (Figure 2a). After incubation, no statistically significant differences (*p* > 0.05) were seen in the survival counts tested in both the nonselective and selected mediums, indicating that all the sublethally wounded cells had been completely repaired. The introduction of inhibitors that target distinct metabolic pathways in the healing of sublethal membrane damage in *E. coli* cells has revealed the biosynthetic requirements for restoring resistance to NaCl in the recovery medium.

In contrast, Figure 2b illustrates that the introduction of penicillin G into the repair medium impedes the recovery process of sublethal injuries in *E. coli* treated with EOCs, including α-terpinyl acetate, α-terpineol, linalool, and citral. This inhibition was evidenced by noticeably slower repair kinetics compared to the TSBYE (Tryptic Soy Broth with Yeast Extract) without any inhibitor (*p* < 0.05). Thus, the repair of these sublethal injuries appears to necessitate peptidoglycan production. However, the presence of penicillin G did not impede the repair of most sublethally damaged cells in *E. coli* culture treated with EOCs such as carvacrol, thymol, *p*-cymene, limonene, and 1,8-cineole (Figure 2b; *p* > 0.05) even after 240 min of repair in the presence of the inhibitor.

### 2.3. Molecular Docking Analysis

Bacterial resistance to β-lactam antibiotics often involves the production of β-lactamases, enzymes that break down the amide group of the β-lactam ring, rendering the antibiotic ineffective. β-lactamases are divided into four classes (A, B, C, and D) based on their amino acid sequence and substrate selectivity [21]. Classes A, C, and D are serine β-lactamases, whereas class B is a zinc-containing β-lactamase. Class A enzymes have a broad substrate profile, are commonly encoded by plasmids, and are easily transferable between cells, thereby compromising clinical antibiotic therapy [22]. These enzymes utilize an active site serine nucleophile to hydrolyze the β-lactam ring on the substrate in a two-step acylation pathway [23]. Among class (A) β-lactamases, Toho-1, which is encoded by a plasmid and produced in *Escherichia coli* TUH12191, was isolated from the urine of a patient treated with β-lactam antibiotics [24].

To understand the binding mechanism of the studied natural compound to the target Toho-1, an in silico theoretical molecular docking approach was used. Fullfitness energy is calculated by considering solvent effects and is therefore a good indicator of binding affinity. α-terpinyl acetate and α-terpineol demonstrated the lowest full fitness scores of −1268.07 and −1265.05 kcal/mol. These molecules occupied 88 and 56 clusters, respectively, within the Toho-1 active site, indicating a favorable binding mode. Furthermore, our results identified two other molecules, linalool and citral, which exhibited fitness scores of −1262.22 and −1257.62 kcal/mol, respectively (Table 1).

To investigate the possible reasons for the differences in the binding energies, we examined the docked complexes using Pymol and Biovia Drug Discovery Studio.

**Figure 3:**
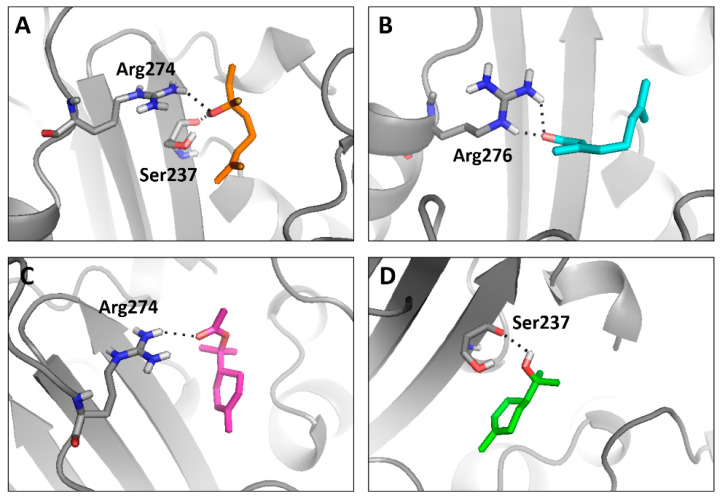
3D diagram of lowest-energy model of Toho-1 complexes. Selected residues at the active site of Toho-1 are marked in sticks; remaining enzyme gray colored cartoon, ligands are in different colors; (**A**) linalool, (**B**) citral, (**C**) α-terpinyl acetate, and (**D**) α-terpineol. The H-bonds are shown as dashed black lines.

**Figure 4:**
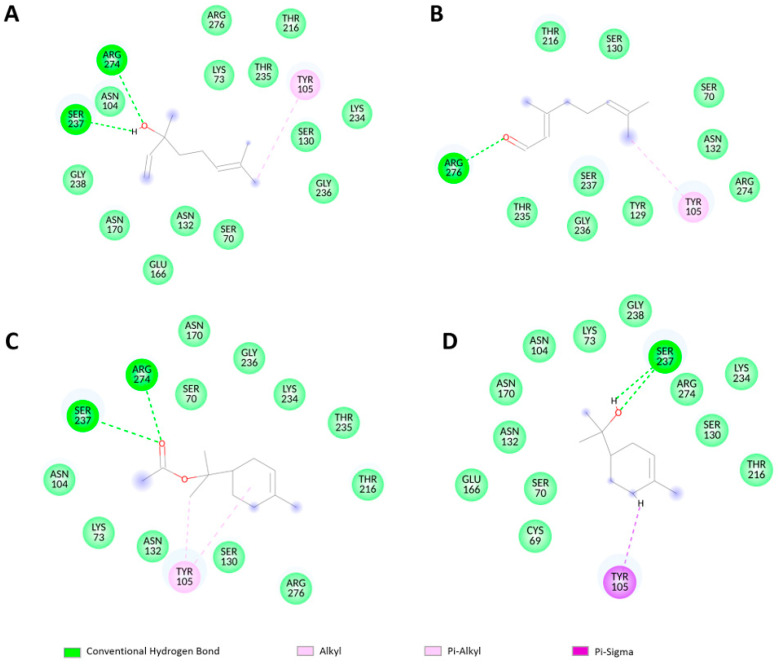
A2D diagram of the intermolecular interactions between ligands and Toho-1 depicted using Discovery Studio Visualizer. Ligands are in different colors; (**A**) linalool, (**B**) citral, (**C**)α-terpinyl acetate, and (**D**) α-terpineol. Residues are differentiated by colors.

A significant number of non-covalent interactions, such as hydrogen bonds and hydrophobic interactions were detected in the four ligands with Toho-1, as shown in Figure 3 and Figure 4. In the Toho1-α-terpinyl acetate complex, there are two hydrogen bonds with Arg274 and Ser237 residues and two hydrophobic interactions with Tyr105. Two hydrogen bonds with Ser237 and one hydrophobic interaction with Tyr105 stabilize the Toho1-Alpha-Terpineol complex.

The Toho1–Linalool complex showed two hydrogen bonds with (Ser237, Arg274) residues and one hydrophobic interaction with Tyr105, whereas one H-bond (with Arg275 residue) and one hydrophobic interaction (with Tyr105 residue) were observed for the Toho1–Citral complex.

Based on the relative docking results, we were able to provide a prioritization list of the suggested constituents. The four major noncovalent binders were α-terpinyl acetate, α-terpineol, linalool, and citral.

## 3. Discussion

In this study, we assessed the impact of the treatment medium pH on the efficacy of antimicrobials in inactivating microorganisms, specifically focusing on essential oil components (EOCs) against *E. coli*. Our investigation considered the effects of pH and the biosynthetic requirements for repairing sublethal damage in bacterial cells. To enhance our understanding, we utilized in silico ligand–protein docking to identify key binding sites and predict the dominant binding modes of ligands with proteins of known three-dimensional structures.

Our findings reveal that antimicrobial agents are particularly effective under acidic conditions, a characteristic that may have been underestimated at neutral pH levels. This aligns with previous research, which attributes this phenomenon to the affinity of Gram-negative bacteria’s outer membrane for hydrophobic elements [25]. Consistent with earlier studies, the minimum inhibitory concentrations (MICs) of EOCs for *E. coli* O157 were found to be 0.2 and <0.2 mg/mL [8].

Despite the critical role of EOCs in food preservation, the biological mechanisms underlying microbial inactivation and resistance to these compounds have not been thoroughly investigated [26]. Further research is necessary to elucidate these mechanisms and optimize the use of EOCs in food safety applications.

The study found that most tested antimicrobials, when acting alone, showed high bactericidal activity at pH 4.0, such as α-terpinyl acetate, α-terpineol, linalool, and citral. Although the hydrocarbons *p*-cymene, (+)-limonene, carvacrol, thymol, and 1,8-cineol, which showed a moderate growth inactivation capacity and were much less effective as bactericidal agents when suspended at pH 7.0, these results have been previously supported by several studies [25,26,27]. On the other hand, this result is in accordance with the extraordinary behavior of α-terpinyl acetate, α-terpineol, linalool, and citral previously described [28,29,30,31,32,33]. According to recent reports, the antimicrobial effects of most EOCs are well known, but their mechanisms of bacterial inactivation for the treatment of cellular damage are not fully understood and still remains a topic of interest for many researchers.

In contrast, when comparing the results of inactivation at pH 7.0, no differences were observed between the spectra of the action of most constituents, except for the effectiveness of α-terpinyl acetate, α-terpineol, linalool and citral as mentioned above. Previous research has found that the pH of the treatment medium is an important environmental component because it affects microbial resistance to physical or chemical inactivants as well as the mode of action of inactivating agents, particularly preservatives [7,29]. The study concluded that oxygenated hydrocarbons and monoterpenes had an effect on the cytoplasmic membrane and exterior wall of Gram-negative bacteria [30], and this might be related to a cell’s hydrophobicity, which is another element to consider when assessing antimicrobial efficacy. Hydrophobia permits EOCs to penetrate the lipids of the bacterial cell membrane, disrupting the structure and resulting in cell permeability. When this happens, the cells release crucial ions and chemicals, potentially leading to cell lysis [31].

In addition, the mechanism of action of EOCs depends on their chemical structure and is not attributable to a unique mechanism but rather a cascade of reactions involving the entire bacterial cell. Most EOCs have a more powerful effect on Gram-positive bacteria than Gram-negative species, likely due to differences in cell membrane compositions [34,35,36,37].

The hydrophobic nature of EOCs allows them to penetrate microbial cells and cause alterations in their structure and functionality. The effects of EOs usually lead to the destabilization of the phospholipid bilayer, the destruction of the plasma membrane function and composition, the loss of vital intracellular components, and the inactivation of enzymatic mechanisms. In some cases, essential oils also alter membrane permeability by destroying the electron transport system [31]. The integrity of the cell membrane is essential for the survival of bacteria because it represents an effective barrier between the cytoplasm and the external environment. When antimicrobial compounds are present in the environment surrounding microorganisms, the bacteria may react by altering the synthesis of fatty acids and membrane proteins to modify the fluidity of the membrane.

In some cases, the antimicrobial activity of EOCs cannot be attributed to a single mechanism but rather involves different biochemical and structural mechanisms at multiple sites within the cell and the cytoplasm.

Hence, some compounds may be beneficial in designing new combined treatments for new applications, especially when they have more appropriate sensory properties, and recent studies have suggested using these natural compounds as novel, efficient antimicrobial agents and produce more targeted treatment strategies to overcome antibiotic resistance such as cephaposperines, which have demonstrated limitations in application due to the actions of ß-lactame inhibitors [38].

This study revealed that EOCs had a definite effect on the presence of sublethal damage to both the cytoplasmic membrane in bacterial cells and the outer membrane in Gram-negative bacteria. Despite the hydrophobicity of cell envelopes induced by the chemical structure of the outer membrane in Gram-negative bacteria and the presence of teichoic acids in the cell wall of Gram-positive bacteria, the majority of cell populations and all the constituents were able to interact with the cell envelopes, resulting in sublethal injuries to most cell populations (N90%). For instance, the results obtained with the major EOCs tested against *E. coli* at pH 4.0 (Figure 1b), or especially with α-terpinyl acetate, α-terpineol, linalool, and citral at pH 7.0 (Figure 1a), illustrate how sublethal damage in the outer membrane occurs faster than in the cytoplasmic membrane, affecting a greater proportion of surviving cells. Sublethal injury, first to the outer membrane and later to the cytoplasmic membrane, appears to drive cells towards inactivation. The evaluation of membrane permeabilization allowed us to relate the existence of inactivated cells (*n* = 90%) after 4 h of incubation in the presence of α-terpinyl acetate, α-terpineol, linalool, and citral, with extensive membrane permeabilization (N90%). These results indicate that the cell envelope is a target in the mechanism of inactivation. However, carvacol, thymol, limonene, *p*-cymene, and 1,8-cineole also caused extensive membrane permeabilization of the *E. coli* cell envelopes, which seemed to be more sensitive at the acidic pH, where considerably less than half of the treated population was luminous while more than 90% of cells were dead [35].

Since cellular envelopes have been shown to play an important role in the inactivation processes of essential oils and their constituents, we have chosen to study the nature of sublethal damage to better understand the structures affected by these elements. Repair of the damaged cytoplasmic membrane in *E. coli* took 4 h and required the synthesis of new proteins and lipids as well as the production of energy. Although energy production is a common prerequisite for repairing cell damage caused by treatments [14,16], the presence of lipids indicates that the membrane plays a crucial role in the inactivation process caused by specific essential oil elements. Several studies have demonstrated that the antibacterial activity of most terpenoids is related to their functional groups. Notably, the hydroxyl group of terpenoid phenolics, as well as the presence of delocalized electrons, contribute significantly to their antifungal action. Carvacrol, for example, outperforms other essential oils like *p*-cymene. This suggests that essential oils and/or their constituents may act on several targets [35].

As demonstrated by Cristani et al., essential oils include a wide range of secondary metabolites that can prevent or impede the development of bacteria, yeast, and mold. They attack a variety of targets, including the membrane and cytoplasm, and in certain situations, they completely alter cell morphology [35].

Regarding the target protein structures implicated in antibacterial action, the present study found numerous non-covalent interactions in four ligands with Toho-1, including the hydrogen bonds and hydrophobic interactions. The Toho1–α-terpinyl acetate complex had two hydrogen bonds with Arg274 and Ser237 residues and two hydrophobic interactions with Tyr105. The Toho1–α-Terpineol complex had two hydrogen bonds with Ser237 and one hydrophobic interaction with Tyr105. The Toho1–Linalool complex had two hydrogen bonds and one hydrophobic interaction. This result supports our in vitro conclusions on the inability to repair sublethal damage to the external membrane of *E. coli* in the presence of the ß-lactamase inhibitor.

## 4. Materials and Methods

### 4.1. Microorganisms and Growth Conditions

The *E. coli* strain used in this study was generously provided by the Pasteur Institute of Algeria. Throughout the investigation, the culture was stored frozen at −80 °C in cryovials. For subculture preparation, a single colony from a plate was inoculated into a test tube containing 5 mL of sterile Tryptic Soy Broth (Biolife, Milan, Italy) (TSBYE) supplemented with 0.6% Yeast Extract (Biolife). The inoculated tubes were then incubated overnight at 37 °C. Subsequently, a 250 mL Erlenmeyer flask containing 50 mL of TSBYE was inoculated with the subculture to achieve a final concentration of 10^4^ cells/mL. The flask was incubated with agitation at 37 °C until a stationary growth phase was attained.

### 4.2. Assessment of Bacterial Inactivation by EOs Constituents

Monoterpene constituents, akin to many constituents found in plant essential oils, are largely immiscible in water. To maintain the integrity of antimicrobial effects and prevent potential damage to cell envelopes, solvents and detergents, which may diminish the antimicrobial potency of essential oils [30], were omitted from these experiments. In agreement with the method described by Friedman et al. [39], initial concentration suspensions were prepared in a citrate-phosphate buffer (McIlvaine buffer) at pH 7.0 (27.09 g/L) and pH 4.0 (23.85 g/L; Hatcher and Bryan [40]) through vortex shaking (Genius 3, Ika, Königswinter, Germany). Stationary phase culture cells were subsequently added to the citrate-phosphate buffers at a final concentration of 2 × 10^7^ cells/mL, with or without each EOCs at a concentration of 0.2 µL/mL. The addition of EOCs did not alter the pH of the buffer. The EOCs treatments were conducted at 20 ± 2 °C for 5 h, with samples extracted at 5 h for the enumeration of survivors. These treatment parameters were selected based on previous studies [16]. Control cells displayed no significant sensitivity when incubated in the citrate-phosphate buffers at pH 7.0 or pH 4.0 for 5 h at 20 °C.

### 4.3. Survival Counts

After treatment, the samples were diluted accordingly to 0.1% *w*/*v* peptone water (Biolife), with 1% *v*/*v* Tween 80 (Biolife) as a neutralizing agent. As a nonselective recovery medium, 0.1 mL of each sample was placed onto tryptic soy agar (Biolife) with 0.6% yeast extract (TSAYE). The plates were incubated for 24 h at 37 °C (*E. coli* strain). However, longer incubation times had no effect on survival. Inactivation was determined by the reduction in log10 counts following treatment.

### 4.4. Measurement of Sublethal Injury

To detect the sublethal damage induced by EOCs, a method involving the plating of survivors after treatment on two distinct types of culture media was used. The first was a nonselective medium, enabling cells to repair sublethal damage and recover, while the second was a selective medium in which survivors were unable to repair their injuries and thus did not multiply [41]. This approach provided insights into protective mechanisms against environmental factors, potentially involving modifications in the bacterial cells’ innate resistance or their ability to heal sublethal injury. To assess cytoplasmic and outer membrane damage following the EOCs treatments, sodium chloride and bile salts were introduced to the nonselective recovery medium. Sensitivity to sodium chloride shows a loss of cytoplasmic membrane osmotic functioning, whereas sensitivity to bile salts suggests a loss of outer membrane integrity and/or impairment of the multidrug efflux mechanisms caused by a loss of proton motive force.

The terms “injury” or “damage” were used to describe the loss of functions partly associated with outer or cytoplasmic membranes. After the EOC treatments, samples were plated onto various media, including TSAYE, TSAYE with 3% sodium chloride (for *E. coli* strains), and TSAYE with 0.35% bile salts (for *E. coli* strains). These concentrations were determined as the maximum non-inhibitory levels for the untreated cells in the stationary phase. Plates containing selective media were incubated for an additional 24 h compared to those with nonselective media, with longer incubation times having no impact on survival counts. The number of sub-lethally injured cells was calculated as the difference between survival counts (cells/mL) on the nonselective medium (TSAYE) and survival counts on the selective media (TSAYE-SC and TSAYE-BS) following the treatments.

### 4.5. Assessment of the Biosynthetic Requirements for Repairing Sublethally Injured Cells

To evaluate the biosynthetic requirements for repairing sublethally injured cells, *E. coli* cells were initially treated with 0.2 µL/mL of each monoterpene, with an initial inoculum size of 2 × 10^7^ cells/mL for 5 h. Subsequently, the treated cells were incubated in sterile TSBYE as a repair medium, either alone or supplemented with penicillin G (10 mg/L), serving as an inhibitor of peptidoglycan synthesis (as reported by Somolinos et al. [14]). The incubation period was set at 37 °C for 4 h. Samples were collected at various intervals, and viable counts were determined using both nonselective and selective media following established protocols. Antibiotic concentration corresponded to the minimum inhibitory growth concentration observed in the native cells.

### 4.6. Protein and Ligands Retrieval and Preparation

**Figure 5:**
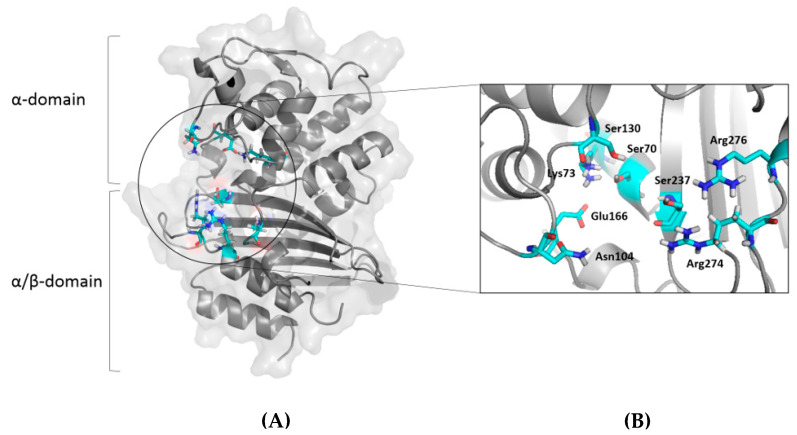
Three-dimensional crystal structure of the class A β-lactamase Toho-1. (**A**) Cartoon and surface representations of Toho-1. (**B**) Stick representations of the active site of Toho-1.

The crystal structure of class A β-lactamase Toho-1 with ID 1IYS was obtained from the Protein Data Bank (PDB), accessed on 21 July 2023 (https://www.rcsb.org/) (Figure 5) [42]. The structural coordinates of the target protein were generated by removing the water molecules, followed by protonation using the Chimera command AddH [43].

We have detailed the binding sites of native Toho-1, as depicted in Figure 5. Toho-1 β-lactamase comprises two highly conserved domains (α and α/β), where the domain interface forms the active site cavity.

Structural data files (SDF) of the nine natural constituents (Table 2) were collected from the PubChem database, accessed on 21 July 2023 (https://pubchem.ncbi.nlm.nih.gov/) [44]. These ligands were optimized using the Open Babel program [45]. For comparative studies, Benzylpenicillin was used as the standard drug. The processed files of the protein and ligands were saved for further computational studies.

### 4.7. Molecular Docking and Cluster Analysis

The molecular docking investigations were carried out using the SwissDock service, accessed on 21 July 2023 (http://www.swissdock.ch/) [46]. The docking operation was performed in an accurate mode, and both the protein and the ligand remained stiff throughout the process. Furthermore, the binding pocket was not specified so as not to interfere with docking to the active site. SwissDock looks for thermodynamically favorable locations for ligand occupancy and generates all the binding modes for each ligand. Only the lowest-energy model was thought to be the most advantageous interaction.

We used the Chimera tool to visualize the generated clusters using the Viewdock command [43]. The lowest energy poses were saved and analyzed using Pymol and Biovia Drug Discovery Studio tools [47]. These tools were used to generate 3D and 2D interaction diagrams, respectively.

### 4.8. Statistical Analysis

The experiments were conducted in triplicate on separate days to ensure reliability and consistency of the results. Inactivation levels were quantified by determining the reduction in log10 counts following each treatment. Error bars in the figures represent the mean value along with the standard deviations derived from a minimum of three independent experiments. Statistical analyses, with a significance level set at *p* = 0.05, were performed using SPSS software version 22 (SPSS Inc., Chicago, IL, USA).

## 5. Conclusions

Our research investigated the bactericidal activity of nine monoterpenes, which are commonly found in the essential oil components (EOCs) of aromatic plants, against *E. coli*. We revealed that these antimicrobials were highly effective at acidic pH levels, whereas their effectiveness would have been overlooked at a more neutral pH. This finding underscores the critical role of pH in evaluating the antimicrobial potential of these compounds.

Our research revealed that EOCs cause sublethal damage to cell membranes under both neutral (pH 7.0) and acidic (pH 4.0) conditions, indicating a direct effect on membrane integrity. A significant aspect of the inactivation process by EOCs involves the sublethal impairment of the *E. coli* cytoplasmic membrane. We found that the addition of penicillin G to the repair medium reduces the recovery of sublethal injuries in *E. coli* exposed to α-terpinyl acetate, α-terpineol, linalool, and citral, suggesting a necessity for peptidoglycan production in repairing these injuries. However, penicillin G did not prevent the recovery process of most sublethally damaged cells treated with other EOCs assessed.

These findings emphasize the need to evaluate phytoactive molecules with antimicrobial activity to develop effective antibacterial agents against antibiotic-resistant strains. Further research is necessary to understand how these antimicrobials interact, whether through synergism, antagonism, or additive effects, and to determine the impact of matrix composition on their activity. This knowledge will help us predict the efficacy of natural essential oils as antimicrobials when used either alone or in combination with other technologies.

## Figures and Tables

**Table 1:** Binding affinities of studied compounds with the β-lactamase Toho-1. The lowest energy model was considered.

Compound Name	Number of Poses at Toho-1 Active Site	ΔG(kcal/mol)	Fullfitness (kcal/mol)
Benzylpenicillin	152	−8.65	−1162.95
Linalool (+)/−	86	−6.13	−1262.22
Limonene (+)−	47	−5.82	−1262.05
Citral	108	−6.43	−1257.62
α-Terpinyl acetate	88	−6.32	−1268.07
α-Terpineol	56	−6.13	−1265.05
Carvacrol	39	−5.85	−1261.54
Thymol	48	−5.99	−1261.30
Eucalypto (1,8-Cineol)	38	−6.05	−1248.12
*p*-cymene	24	−5.75	−1251.82

**Table 2:** Natural constituents with PubChem identifiers and their respective descriptions.

Compound Name	Pubchem CID	Description
Benzylpenicillin	5904	Natural penicillin
Linalool (+)/−	6549	Plant metabolite
Limonene (+)−	22311	Natural product found in plant and other organisms
Citral	638011	Plant metabolite
α-Terpinyl acetate	111037	Natural product found in plant and other organisms
α-Terpineol	17100	Natural product found in plant and other organisms
Carvacrol	10364	Phenol derivative of cymene. Antimicrobial agent, anti-fungal agent, food additive.
Thymol	6989	Phenol derivative of cymene with antiseptic, antibacterial, and antifungal actions,
Eucalypto (1,8-cineole)	2758	Natural product with anti-inflammatory proprieties. Flavoring agent
*p*-cymene	7463	Plant metabolite

## Data Availability

The data presented in this study are available in this article.
